# Comparative epigenomics to clinical trials in human breast cancer and canine mammary tumor

**DOI:** 10.1080/19768354.2025.2477024

**Published:** 2025-03-19

**Authors:** Su-Jin Jeong, Kang-Hoon Lee, Je-Yoel Cho

**Affiliations:** a Department of Biochemistry, College of Veterinary Medicine, Research Institute for Veterinary Science, and BK21 PLUS Program for Creative Veterinary Science, Seoul National University, Seoul, Republic of Korea; b Comparative Medicine Disease Research Center, Seoul National University, Seoul, Republic of Korea

**Keywords:** Epigenetics, breast cancer, canine mammary tumor

## Abstract

Epigenetics and epigenomics are captivating fields of molecular biology, dedicated to the exploration of heritable alterations in gene expression and cellular phenotypes, which transpire devoid of any discernible modifications to the fundamental DNA sequence. This intricate regulatory apparatus encompasses multiple mechanisms, prominently featuring DNA methylation, histone modifications, and the involvement of non-coding RNA molecules in pivotal roles. To achieve a comprehensive grasp of these diverse mechanisms, it is imperative to conduct research employing animal models as proxies for human studies. Since experimental animal models like mice and rats struggle to replicate the diverse environmental conditions experienced by humans, this review focuses on comparing common epigenetic alterations in naturally occurring tumors in canine models, which share the human environment, with those in humans. Through this, we emphasize the importance of an epigenetic regulation in the comparative medical approach to a deeper understanding of cancers and further development of cancer treatments. Additionally, we elucidate epigenetic modifications pertinent to specific developmental stages, the ageing process, and the progression of various diseases.

## Introduction

The past decade has seen a rapid emergence of epigenetics as a significant contributor to carcinogenesis (Bae and Kim 2008; Kristensen et al. 2009; Taby and Issa 2010; Campbell and Turner 2012; Lu et al. 2020; Jang et al. 2023). Aberrations in the normal DNA methylation patterns and histone modifications have been recognized as targets for therapy, which, unlike conventional chemotherapy, aims to revert the abnormal state of malignant cells to a more normal condition (Tandon et al. 2016; Lu et al. 2020; Buocikova et al. 2022a). For this purpose, current epigenetic drugs are used to target specific enzymes or proteins involved in epigenetic modifications, such as DNMTs (DNA methyltransferases) and HDACs (histone deacetylases) (Luker et al. 2020; Bondarev et al. 2021).

Experimentally, tumors have been tested in laboratory animals like rodents, facilitating newer insights into the diagnostic and therapeutic arena (Blewitt and Whitelaw 2013). Nonetheless, their current utility in developing epigenetic cancer drugs is somewhat limited compared to their role in studying other aspects of cancer biology (Hunter 2012). Firstly, many epigenetic drugs targeting epigenetic modulators, especially DNA methylase or histone deacetylase inhibitors, do not exhibit locus-specificity in their action, causing broad changes in gene expression (Blewitt and Whitelaw 2013). Animal models may not provide additional insights into this aspect of drug action, as these drugs typically affect genes similarly in animals and humans (Brodey 1979; Pinho et al. 2012; Liu et al. 2014; Herranz et al. 2016; Abdelmegeed and Mohammed 2018; Gray et al. 2020). In addition, there can be significant genetic, epigenetic, and physiological differences between animal models and humans. These differences limit the predictive value of animal studies for human responses to epigenetic drugs and can profoundly affect the epigenetic alternations, like the laboratory setting and use of inbred strains (Hunter 2012).

The canine model is highly valuable in human medicine due to its genetic, epigenetic, and physiological similarities with humans (Abdelmegeed and Mohammed 2018). Canines naturally undergo many diseases, such as tumors, cardiovascular diseases, autoimmune disorders, and neuronal disease, which closely mimic human pathophysiology (Pinho et al. 2012; Abdelmegeed and Mohammed 2018). Epigenetic regulation of canines shares key features with humans, making them an excellent model for studying how environmental factors influence gene expression and disease progression. Unlike rodents, canines have a longer lifespan and a complex immune system, allowing for more accurate evaluation of immunotherapies, including cancer treatments and vaccines (Park et al. 2016). Metabolic and pharmacokinetic profiles of canines also resemble humans, making them useful for drug development and pharmacological testing (Wittenburg et al. 2010; Fulkerson et al. 2017). In comparative oncology, canine tumors exhibit molecular and histopathological similarities to human malignancies, facilitating more efficient and clinically relevant pre-clinical evaluation of emerging treatments (Elshafae et al. 2017; Abdelmegeed and Mohammed 2018). Certain canine breeds possess a strong hereditary susceptibility to specific conditions, providing a valuable model for investigating genetic risk factors and precision therapies (Rowell et al. 2011). Additionally, canines share the same environment as humans, making them useful for understanding the effects of environmental carcinogens on health (Owada et al. 2024). Their role in stem cell therapy and regenerative medicine is expanding, particularly in orthopedics and spinal cord injury research. Overall, the translational potential of canine models accelerates the development of personalized treatments, benefiting both human and veterinary medicine (Rowell et al. 2011; Cekanova and Rathore 2014; Zeng et al. 2020).

The canine model presents notable advantages over the rodent model in epigenetic research due to its closer alignment with human epigenetic mechanisms (Wang et al. 2020a; Son et al. 2023). Compared to rodents, canines and humans share more conserved patterns of CpG islande density, chromatin landscapes, and non-coding RNA regulation, making canines a more relevant model for studying epigenetic therapies (Han and Zhao 2009; Le Béguec et al. 2018; Son et al. 2023).

Unlike rodents, which often require genetic engineering or artificial disease induction, canines naturally develop conditions influenced by epigenetic alterations over time (Pinho et al. 2012). This spontaneous disease development allows for a more realistic examination of disease progression and response to epigenetic interventions (Pinho et al. 2012). Additionally, the longer lifespan of canines provides a better framework for studying age-related epigenetic changes and their role in chronic diseases – something that is challenging to replicate in short-lived rodent models (Wang et al. 2020a).

Another key advantage is that, unlike rodents raised in controlled laboratory environments, canines share the same living conditions as humans (Owada et al. 2024). Their exposure to similar dietary patterns, lifestyle factors, and environmental pollutants creates a more representative model for studying how external factors influence epigenetic modifications (Owada et al. 2024).

In comparative oncology, canine cancers exhibit epigenetic alterations akin to those seen in human cancers, including widespread DNA hypomethylation and promoter hypermethylation of tumor suppressor genes (Jeong et al. 2019; Nam et al. 2020; Schabort et al. 2020; Nam et al. 2023). This similarity makes canines an ideal model for evaluating epigenetic therapies such as DNA methylation inhibitors and histone deacetylase inhibitors.

Furthermore, the canine immune system bears a greater resemblance to the human immune system than that of rodents, particularly in how epigenetic modifications regulate immune responses and inflammation (Park et al. 2016). This similarity is crucial for studying immune-related epigenetic therapies, including checkpoint inhibitors for cancer treatment (Park et al. 2016). Hence, genome-wide methylation studies of PBMC revealed that altered DNA methylation patterns are hallmarks of tumor progression as well as changes in the composition and function of the global immune landscape in both canines and humans (Jeong et al. 2019; Nam et al. 2023; Wang et al. 2023).

Taken together, these factors make the canine model a more relevant and translationally valuable system for understanding human epigenetics and developing targeted therapeutic strategies.

**Figure 1.**
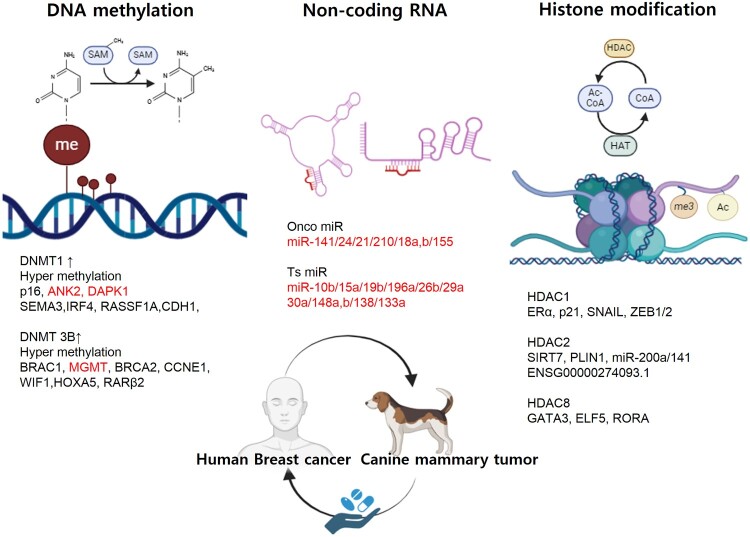
Schematic overview of epigenetic alterations in human breast cancer and canine mammary tumor. Human breast cancer is associated with aberrant DNA methylation, histone modifications and non-coding RNAs expressions. Given the broad similarities between canine mammary tumors and human breast tumors, particularly in terms of epigenetics, canine mammary tumor patients are increasingly recognized as valuable models for both comparative oncology and the development of epigenetic drugs (epi-drug).

In this review, we compare the epigenetic changes observed in canine mammary tumors (CMTs) and human breast cancers (HBCs), highlighting the potential of these studies to evaluate the efficacy and safety of epigenetic drugs. These comparisons suggest that such models may help bridge the gap between pre-clinical findings and human clinical applications, as summarized in Figure 1.

## 1. Epigenetics in human breast cancer and canine mammary tumor

### 1) DNA methylation

In the promoter domain, hypermethylation of CpG islands often results in gene silencing, including tumor suppressor genes, while hypomethylation can contribute to genome instability and up regulation of onco genes (Kristensen et al. 2009). Since this is closely related to tumorigenesis and occurs before gene mutations, the DNA methylation pattern acts as a potential biomarker for cancer diagnosis, prognosis, and therapeutic targets, providing insight into the complex molecular mechanisms of cancer incidence and progression (Chen and Wu 2016). This section shows the methylation of various molecular markers that have been reported in relation to breast cancer.

#### Breast cancer-associated genes

The BRCA1/2 genes are regarded as tumor suppressor genes (TSG) that encode proteins involved in DNA repair mechanism, cell cycle regulation, maintenance of genome stability and physiological pathways (Wei et al. 2005; Lobanova et al. 2021). BRCA1/2 expression is reduced in various cancers, including sporadic HBC (Wei et al. 2005). Promoter DNA methylated of BRCA1 ranges from 14.3% to 29.8% and is common in high-grade, ER-, PR- patients and more commonly exhibits in the sporadic basal-like HBC (Wei et al. 2005; Turner et al. 2007). In another study, BRCA1/2 is methylated 34% and 50%, respectively, and 20% of all cases show both methylated (Ruscito et al. 2021). The portion of methylated BRCA2 was slightly higher in those not treated with neoadjuvant therapy than HBC patients who received neoadjuvant therapy (Lobanova et al. 2021).

The association between BRCA1/2 downregulation and CMT development has been reported (Nieto et al. 2003; Yoshikawa et al. 2015; Qiu and Lin 2016). While the expression level of BRCA1 was particularly reduced in malignant CMTs, the promoter methylation of the 5’ flanking region had no correlation with expression (Qiu and Lin 2016; da Costa Ferreira et al. 2019).

O6-methylguanine-DNA methyltransferase (MGMT) is a widely expressed gene involved in the repair of DNA damage (Gerson 2004). MGMT is responsible for removing alkylating adducts from the DNA molecules, preventing G to A transition mutations or strand breaks in DNA within pre-malignant lesions (Gerson 2004). MGMT is silenced in HBC via epigenetic modification. In a meta-analysis conducted by An et al., the promoter methylation of MGMT was found to be statistically higher in HBC patients compared to non-tumorous samples (An et al. 2017).

DAPK1 methylation and protein levels were assessed using MSP and immunohistochemistry (*n* = 128) as well as western blotting (*n* = 56). Among 128 tumor samples, 62.2% of cases with DAPK1 methylation exhibited reduced or weak protein expression, while 75.8% of unmethylated cases showed strong protein expression (Ren et al. 2018). These findings revealed a significant inverse correlation between DAPK1 promoter hypermethylation and protein level. The hypermethylation and reduced protein expression of DAPK1 were correlated with hormone receptor negativity (Asiaf et al. 2019).

Both MGMT and DAPK1 genes are considered TSG in both HBC and CMT (An et al. 2017; Ren et al. 2018; Yadav et al. 2018). MGMT and DAPK1 methylation was assessed in CMT cell lines (CHMm and CHMp), CMT tissues and blood from 24 female canines by methylation-specific PCR (MSP). In CMT blood samples, DAPK1 and MGMT were completely methylated in 37.5% (9/24) of cases and partially methylated in 54.17% (13/24). In malignant tissue, 69.23% (9/13) were completely methylated (Asiaf et al. 2019). Following treatment with 5-azacytidine in CMT cell lines, the expression of these genes increased, resulting in reduced methylation levels and decreased cell proliferation compared to the control (Ren et al. 2018).

#### miRNAs

Promoter hypermethylation of microRNA was also conserved in both humans and canines. MiR-34c, known to increase cisplatin sensitivity and suppress metastasis and epithelial–mesenchymal transition (EMT), was hypermethylated in both HBC and CMT patients (Yu et al. 2012; Jeong et al. 2019). Similarly, miR-124-2, which is hypermethylated, inhibits multiple pro-metastasis-related genes, including CTGF, RAS homolog gene family member G (RHOG), ITGB1, and ROCK1 (Lan et al. 2018; Jeong et al. 2019). The promoter of miR-149 is also hypermethylated in both HBC and CMT. It can restrict M2 polarization of macrophages by targeting colony-stimulating factor-1, reducing lung metastases and impairing M2 macrophage infiltration of primary tumors (He et al. 2014; Jeong et al. 2019). However, miR-184, which is known as a putative tumor suppressor and regulates the AKT/mTORC1 pathway by targeting AKT2, TSC2, and PRAS40, is found to be hypermethylated in HBC but not in CMT (Park et al. 2017).

#### Non-genomic regions

Non-promoter DNA methylation containing transcription factor (TF) binding motifs and sequence repetitive transposable DNA elements such as Alu and long interspersed nucleotide element-1 (LINE-1) DNA methylation altered in both HBC and CMT (Lee et al. 2019). The research conducted by Lee et al., identified that global LINE-1 Two open reading frame (ORF) regions, ORF1 and ORF2, are well conserved, observed in both canine and humans. The relative cell-free DNA concentration of LINE-1 methylation in canines is significantly lower in both benign and malignant CMT compared to healthy control groups. This suggests the potential of LINE-1 methylation as a diagnostic marker for CMT (Lee et al. 2019).

These results suggest that promoter methylation as well as non-promoter methylations, including LINE-1 also aberrantly methylated in both humans and CMT (Lee et al. 2019; Ponomaryova et al. 2020). From recent advancements in canine research, global DNA methylation quantification, assessed through immunostaining, has been identified as a distinguishable tool between benign and CMT (*p* = 0.024) (Biondi et al. 2021). Global hypomethylation patterns are more prevalent in malignant tumors with higher relapse behavior (*p* = 0.011) as well as larger tumor size (*p* = 0.028). This suggests that global hypomethylation is associated with genomic instability and the risk of malignancy in CMT, similar to its human counterpart (Biondi et al. 2021).

As expected, DNA methylation changes in both genomic and non-genomic regions are shared in humans and CMT patients, providing valuable perspectives for mechanistic studies in both species. The canine genome has been reported to have a very close promoter-associated CpG island density (160.7/Mb) in humans (156.8/Mb) than those mice (118.4/Mb) (Han and Zhao 2009). Furthermore, due to the stability of DNA methylation, it can serve as an applicable biomarker for the early detection of HBC as well as CMT (Lee et al. 2019; Wang et al. 2023). However, some differences still exist between humans and canines, such as the promoter methylations status of BRCA1/2 genes. Additional studies with large cohorts focusing on DNMT expression as well as the methylation status of BRCA1/2 promoters are needed to identify prognostic value in CMT patients.

### 2) Histone modification

HBC cells with distinct characteristics display different histone modification patterns, including the loss of chromatin marks like H3K27me3 (Hsieh et al. 2020). Histone acetylation also plays a significant role in HBC development, with histone acetyltransferases (HAT) affecting gene expression and inhibiting cell survival (Elsheikh et al. 2009). Inhibitors of histone deacetylases (HDAC) and HAT have emerged as potential options for cancer therapy, particularly in HBC treatment (Shin et al. 2025). On the other hand, histone methylation can lead to reversible gene suppression, and DNA methylation may follow as a secondary event, resulting in long-term gene repression (Varier and Timmers 2011; Campbell and Turner 2012).

**Figure 2.**
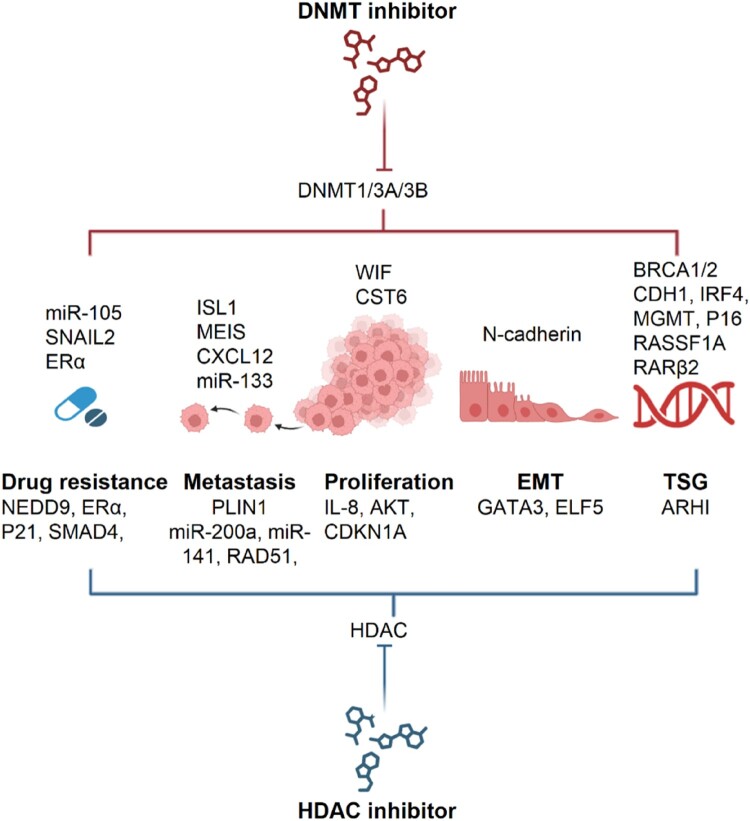
Roles of DNA methyltransferase (DNMT) and Histone deacetylase (HDAC) in tumorigenesis. DNMTs and HDACs regulate the fine-tuning of various molecules, consequently playing a role in multiple steps of tumorigenesis. Epi-drug, including DNMT inhibitor (DNMTi) and HDAC inhibitor (HDACi) targeted histone modifiers. As a result of DNMTi or HDACi, exposures inhibited multiple tumorigenic pathways.

Pivotal roles in tumorigenesis of imbalanced histone modifiers have been reported (Campbell and Turner 2012). In HBC, HDAC is responsible for ERα and ER target as well as tumor-associated genes activation and is involved in tumorigenic or tumor suppressive functions (Gryder et al. 2013) (Figure 2).

Alterations of histone marks of global or regulatory regions of specific genes involved in tumorigenesis as well as subtype specifications. For instance, in 880 HBC samples, seven histone marks (H3K9ac, H3K18ac, H4K12ac, H4K16ac, H3K4me2, H4K20me3, and H4R3me2) exhibited a negative correlation with tumor grade (Elsheikh et al. 2009). Notably, elevated H4R3me2 and H3K9ac levels were detected in lower lymph node stages, while H4R3me2, H3K9ac, and H4K16ac were reduced in larger tumor sizes. Significantly, a prevalent absence of H4K16ac, often found in these specimens, suggests its potential as an early event in HBC development, serving as a diagnostic marker. Conversely, triple-negative breast cancer (TNBC) and HER2-positive HBC displayed diminished levels of these markers, indicative of their poorer prognosis (Elsheikh et al. 2009). Xi et al. examined 5 core histone marks (H3K4me1/3, H3K27me3, H3K9me3 and H3K36me3) in 5 subtypes of HBC (ER, luminal A/B, TNBC claudin-low, TNBC basal-like and HER2) (Xi et al. 2018). Through 234 ChIP-seq datasets obtained from a public database and identified unique chromatin patterns specific to each subtype (Xi et al. 2018). These patterns revealed distinct active epigenetic marks in estrogen signaling and ERK/MAPK pathways, particularly between hormone-responsive subtypes and TNBC (Xi et al. 2018). In the case of the repressive mark, H3K27me3 more distinctive histone mark than H3K9me3 and is highly enriched in NOD-like signaling pathways across the caner types (Elsheikh et al. 2009). Interestingly, AFAP1-AS1 was identified as a TNBC-specific active histone-enriched gene and potentially regulates mesenchymal markers, suggesting that it may promote the migration and proliferation of TNBC (Xi et al. 2018). H3K27ac, regarded as an enhancer histone mark, also involved transcription activation (Li et al. 2019). Hyper activation in enhancers is one of the epigenetic features in tumorigenesis. Li et al. described enhancer histone marks, H3K27ac and H4K8ac, increased in cancer and identified 148 super-enhancers, co-localized with H3K4me3, and revealed highly expressed in tumor (Li et al. 2019).

There is still a lack of genome-wide histone modification analysis in CMT. It was reported that according to RNA-seq and Immunohistochemical (IHC) analysis of 12 CMT patients, 35 chromatin modification genes and repressive histone mark H3K9me3 were down regulated (Liu et al. 2014). Conversely, abnormal H4 acetylation was highly enriched in complex mammary tumors. Particularly within complex carcinomas, there are minimal genomic copy number alterations in their sequence (Liu et al. 2014). These tumors exhibit low rates of sequence mutation, underscoring the prominence of epigenetic alterations in this tumor type (Liu et al. 2014). Vinothini et al. described that HDAC1 expression increases according to severity in CMT (Vinothini et al. 2009). HDAC1, known to be significantly increased in HBC patients, negatively regulates ERα expression and is thus considered a potential therapeutic target for ER-negative patients. According to Choi et al., EZH2 expression was estimated by IHC and identified its expression associated with malignancy in CMT, similar to its role in humans (Campbell and Turner 2012; Choi et al. 2016).

Research has shown that combining HDAC inhibitors with demethylating agents or physical therapies can simultaneously affect DNA methylation and histone modification, revealing synergistic effects in certain HBC cell types. Functional studies have identified interactions between DNA methylation and histone modification involving various genes, ultimately contributing to the silencing of tumor suppressor genes in HBC cells.

### 3) Non-coding RNA

In recent years, non-coding RNAs (ncRNAs) have emerged as pivotal roles in the intricate landscape of cellular processes as well as tumorigenesis. NcRNAs are classified by size into miRNA, lncRNA, circular RNA and piwi RNA devoid of protein-coding capacity. They play a significant role in gene expression modulation through epigenetic mechanisms (Taft et al. 2010). In this review, we concentrate on the most extensively studied miRNAs and lncRNA concerning their relevance to HBC and CMT (Table 1).

**Table 1.** Aberrantly expressed ncRNAs and their target.

NcRNA	Function	Target	Expression in human	Expression in canine	Reference
miR-18a/b	Proliferation	IL-1β, IL-6, TNFα and ERα	Increased	Increased	(Leivonen et al. 2009; Jiménez-Garduño et al. 2017; Fish 2019; Egeland et al. 2020; Manore et al. 2022; Abbate et al. 2023)
miR-19	Drug resistance	PTEN	Increased	Increased	(Liang et al. 2011; Li et al. 2018; Fish et al. 2020)
miR-21	Proliferation Invasion	N-cadherin, Vimentin, α-SMA PDCD4, TPM1 and mapsin	Increased	Increased	(Zhu et al. 2008; Han et al. 2012; Ramadan et al. 2022)
miR-155	Proliferation	SOCS1 and C/EBPβ	Increased	Increased	(Zhang et al. 2018; Zuo et al. 2018; Kim et al. 2023)
miR-141	Drug resistance	ERBB4 and INK4a	Increased	Increased	(Lutful Kabir et al. 2015; Wang et al. 2017; Han et al. 2019)
miR-133a	EMT	EGFR	Decreased	Decreased	(Hua et al. 2021; Chen et al. 2022)
miR-133b	Metastasis	SOX9	Decreased	Decreased	(Wang et al. 2018; Chen et al. 2022)
miR-124	Metastasis	CTFG, RHOG, ITGB, ROCK	Decreased	Decreased	(Lv et al. 2011; Liang et al. 2013; Ren et al. 2022)
miR-497	Proliferation Invasion	Hippo signaling, PTEN	Decreased	Decreased	(Shen et al. 2012; Cheng et al. 2018; Zhang et al. 2021)
HOTAIR	Migration, invasion, metastasis	miR-601	Increased		(Le Béguec et al. 2018; Wang et al. 2020c)
NEAT1	EMT, metastasis	ZEB1	Increased		(Zhang et al. 2017; Jiang et al. 2018; Le Béguec et al. 2018; Knutsen et al. 2022)

ncRNA: Non-coding RNA; IL:Inter leukin; ER: Estrogen receptor; PTEN: Phosphatase And Tensin Homolog; PDCD: Programmed Cell Death 4; TPM1: Tropomyosin 1; SOCS1: Suppressor Of Cytokine Signaling 1; ERBB4: Erb-B2 Receptor Tyrosine Kinase 4; EGFR: Epidermal Growth Factor Receptor; SOX9: SRY-Box Transcription Factor 9; RHOG: Ras Homolog Family Member G; RHOG: Rho Associated Coiled-Coil Containing Protein Kinase; ITGB: Integrin Subunit Beta; ROCK:Rho Associated Coiled-Coil Containing Protein Kinase; ZEB1: Zinc Finger E-Box Binding Homeobox 1.

#### Micro RNAs

MicroRNAs (miRNA), short non-coding RNAs consisting of 19–25 nucleotides, modulate gene expression by binding to motifs within the 3’ untranslated region (UTR) of mRNA. Over 60% of mRNAs are thought to harbor miRNA target sites in their 3’ UTR region, indicating significant regulation in both normal and diseased conditions including cancers (Zhao et al. 2011). Cancer-associated miRNAs can be categorized into two groups: tumor suppressor miRNAs (tsmiRs) and Oncogenic miRNAs (oncomiRs) (Loh et al. 2019). OncomiRs are up-regulated in cancers, promoting proliferation, metastasis and angiogenesis. TsmiRs can target oncogenes involved in tumorigenesis or inhibit the tumor-associated features above described. TsmiR and oncomiR are extensively reviewed in (Mulrane et al. 2013; Jeong et al. 2019; Loh et al. 2019).

##### MiR-18a/b

MiR-18a/b, specifically, show increased expression in highly proliferative HBC and are associated with a negative prognosis in ER-negative (ER-) HBC (Egeland et al. 2020). In ER- HBC, miR-18a and miR-18b play a role in increased cytokine expression, including IL-1β, IL-6, and TNFα, contributing to a pro-inflammatory response (Egeland et al. 2020). In the context of a CMT cohort study, it is indicated that extracellular vesicle-derived miR-18a/b was increased in CMT and may be correlated with ERα, as supported by remarkably high miRDB scores (Fish 2019). In their human counterpart, miR-18a also directly regulate ERα by targeting the 3’ UTR region (Leivonen et al. 2009; Abbate et al. 2023).

##### MiR-19b

Another oncogenic microRNA, miR-19b, is overexpressed in HBC tumor tissue and cell lines (MCF7 and MDA-MB-231) and is associated with poor overall survival in HBC patients (Li et al. 2018). MiR-19b also has been reported to accelerate tumor progression and enhance multidrug resistance via loss of PTEN, leading to PI3K/AKT activation and consequently activating multidrug resistance-related genes such as MDR-1, MRP1, and BCRP (Liang et al. 2011; Li et al. 2018). However, the detailed mechanism remains undisclosed.

##### MiR-21

MiR-21 is up-regulated in both canine and human cancers. In HBC, miR-21 enhances tumor cell proliferation, and metastasis and inhibits apoptosis by targeting various TSGs (Zhu et al. 2008). In HBC, treatment with antagomiR-21 led to decreased levels of N-cadherin, α-SMA, and Vimentin, demonstrating a reduced epithelial–mesenchymal transition (EMT) phenotype. Furthermore, miR-21 promotes tumor invasion and metastasis by directly targeting PDCD4, mapsin and TPM1 (Han et al. 2012). In canines, miR-21 up-regulated 12.84-fold in serum level in all CMT samples compared to normal levels (Ramadan et al. 2022).

##### MiR-155

MiR-155 is another miRNA increased in CMT and HBC (Zhang et al. 2018; Zuo et al. 2018). In HBC, it is associated with cancer cell proliferation, invasion and angiogenesis by targeting the suppressor of cytokine signaling 1(SOCS1) (Zhang et al. 2018; Zuo et al. 2018). MiR-155 mediated C/EBPβ reduction makes it more sensitive to TGF-β1 induced EMT transition in TGF-β1 in HBC. In canines, a miRNA microarray was conducted on 20 tissue samples, revealing an increase in miR-155 in canine ductal carcinoma tissue, with the predicted target SOCS1 being downregulated (Kim et al. 2023).

##### MiR-141

The function of miR-141 is still controversial, it is associated with not only poor outcomes but also involved in the gain of trastuzumab resistance, which is mediated by ERBB4 (Han et al. 2019). MiR-141 expression increased in docetaxel-resistant HBC cell lines, regulating the transcription of eIF4E, a factor known to decrease HBC cell growth (Wang et al. 2017). In canines, miR-141 is recognized as an oncomiR that regulates post-transcriptional silencing by targeting the 3’ UTR of the INK4A gene. The INK4A gene is acknowledged as a tumor suppressor, playing a crucial role in regulating the cell cycle process (Lutful Kabir et al. 2015).

##### Mir-133a/b

As a tsmiR, mir-133a was involved in various cellular processes such as proliferation, epithelial-to-mesenchymal transition (EMT) and drug sensitivity in various cancer (Hua et al. 2021). This miRNA suppressed cell cycle and proliferation by targeting EGFR. Additionally, miR-133 might inhibit UCP2 in the MCF7 cell line, consequently sensitizing doxorubicine resistance (Hua et al. 2021).

MiR-133b regarded as tsmiR, was down regulated in many kinds of cancers including HBC (Wang et al. 2018). Mir-133b could affect HBC metastatic potential by targeting SOX9 in HBC (Wang et al. 2018). Mir-133b expression was also reduced in CMT cell lines. According to miRNA expression profiling of CMT conducted by NGS, the miR-133 family was down regulated in all types of tissues assayed, including adenoma, benign mixed, and adenocarcinoma (Chen et al. 2022).

##### MiR-124

In the human cohort, miR-124 is highly expressed in the nervous system and is reduced by DNA methylation in various cancers (Lv et al. 2011). As a tsmiR, miR-124 regulates multiple steps of metastatic potential in highly aggressive HBC cells by directly targeting prometastatic genes, including CTFG, RHOG, ITGB1, and ROCK1 (Lv et al. 2011). Additionally, overexpression of miR-124 strongly inhibits cell migration and EMT transition by regulating the EMT-related transcription factor SLUG. Consistent with the effect of overexpressed miR-124, the protein level of the epithelial marker increased, whereas the mesenchymal marker decreased (Liang et al. 2013). MiR-124 is also down regulated in CMT tissues and cell lines compared to normal specimens. This study identified that mir-124 inhibits CMT cell proliferation, invasion, and migration by targeting 3’ UTR of CDH2 mRNA at bases 241–247 (Ren et al. 2022).

##### MiR-497

In HBC, miR-497 is regarded as a tsmiR that induces apoptosis and inhibits proliferation and invasion. Mir-497 directly binds Bcl-w and increases the apoptotic rate of MCR7 cell lines (Shen et al. 2012). Li et al identified that YAP1, as a target of miR-497, is a downstream molecule of the Hippo signaling pathway, which is involved in HBC growth inhibition by negatively regulating PTEN (Cheng et al. 2018). MiR-497 significantly down regulated in CMT tissues compared to normal samples. Overexpression of miR-497 reduced cell proliferation, migration ability and increased apoptotic rate compared to control cells. MiR-497 directly targets IRAK2, resulting in high expression of Caspase-3, BAX, and PARP in CMT cells (Zhang et al. 2021).

Zhou et al. (2008) identified 357 canine miRNAs, among which 300 are conserved between humans and canines (Zhou et al. 2008). Studies on CMT miRNAs have shown that expression patterns, related pathways, and clinical characteristics are highly conserved in both canines and their human counterparts (Zhou et al. 2008). Therefore, these miRNAs offer promising anti-cancer strategies and serve as excellent targets for the treatment of HBC and CMT.

##### Long non-coding RNA

LncRNAs, exceeding 200 nucleotides, can directly interact with chromatin, and histone-modifying enzymes and function as miRNA sponges, reducing their regulatory effect (Taft et al. 2010). Representatively, HOTAIR is one of the significantly increased lncRNA in HBC tissues and cell lines. HOTAIR expression correlated with poor prognosis as well as increased proliferation, invasion, and migration abilities. Mechanistically, HOTAIR directly targets miR-601 known as tsRNA followed by increased pAKT/AKT (Wang et al. 2020c). Another lncRNA, NEAT1, is one of the well-studied oncogenic lncRNA in HBC (Zhang et al. 2017; Knutsen et al. 2022). NEAT1 can be sponging miR-448, consequently increasing the expression of ZEB1, which plays a role in epithelial–mesenchymal transition (EMT) and metastasis (Jiang et al. 2018). Although rapid sequence turnover of lncRNAs during the evolutionary process, Béguec et al. identified 900 conserved lncRNAs between humans and canines and the authors found that 26% of human and canine orthologous lncRNAs were expressed in identical tissues in both species, suggesting their involvement in the evolutionary process (Le Béguec et al. 2018). In canines, 939 synthetic lncRNAs are conserved between canines and humans. Interestingly, canine lncRNA overlapped transposable element (TE) regions (Le Béguec et al. 2018). These results suggest that evolutionarily conserved lncRNAs might serve as useful biomarkers or therapeutic targets in both species. Furthermore, unique lncRNA resources reveal an unveiled mechanism of species specificity at a molecular level. However, there is still lack of extensive comparative data analysis is still needed for predicting lncRNAs with clinical relevance in HBC and CMT.

## 2. Epigenetics in animals

### 1) Animal models

Cancer research is a dynamic and critical field that relies on the use of animal models to better understand the complex mechanisms underlying cancer development and to evaluate potential therapeutic targets. Mouse is a commonly employed laboratory animal in cancer research (Zeng et al. 2020). Under laboratory conditions, the genome and the environmental conditions of mice can be strictly controlled, and this became the basis of epigenetic studies.

Since mutations in most epigenetics modulators cause lethal, before 2015, haplo-insufficient mice were mainly used in mouse DNA methylation studies (Blewitt and Whitelaw 2013; Niles et al. 2013; Kemp et al. 2014). The hypomorphic allele of DNMT1 resulted in a global loss of DNA methylation, particularly contributing to the development of T-cell lymphoma, suggesting that global hypomethylation also involved lymphoma tumorigenesis. In the APCMin/wt mouse model, DNMT3B/MBD2 double mutant mice inhibited intestinal adenoma progression (Espada and Esteller 2013). In MYC-induced lymphoma mice, DNMT3B hypomorphism increased cell proliferation and accelerated tumor growth by up-regulating the expression of tumor modifier genes. In MYC-induced lymphoma mice, conditional knock down of DNMT3B increased cell proliferation and accelerated tumor growth by up-regulating the expression of tumor modifier genes (Vasanthakumar et al. 2013).

In 2015, a TALE-mediated epigenetic suppression method was developed, which utilized the catalytic domain of DNMT to selectively bind and induce methylation in the target gene (Bernstein et al. 2015). Then, in 2016, a combined method of the TET1 catalytic domain and CRISPR/Cas9 system was developed for inducing site-specific DNA hypomethylation (Morita et al. 2016).

Rodent-based classical studies prove beneficial in leveraging insights following the discovery of genes undergoing epigenetic changes in cancer research. However, the identification of novel causative genes and their parallel integration with human clinical trials remain unattainable. Furthermore, laboratory models do not reflect the effect of environmental carcinogens.

### 2) Companion animals

On the other hand, companion animals have been highlighted due to their similarities with human cancers. Canines develop spontaneous tumors, inflammatory bowel disease (IBD), and diabetes, which have similar histopathological features, genomic alterations, and environmental risk factors (Cerquetella et al. 2010; Cekanova and Rathore 2014; Adin and Gilor 2017). The canine model has numerous advantages compared to mice, including the size, anatomical and metabolic similarities to humans facilitating the application of surgical procedures and drug pharmacokinetics (Martinez et al. 2020).

CMT is the most frequent and naturally occurring malignant disease in female canines with a lifetime risk ranging from 23% to 34%. Female macaques, including non-human primates (NHP) model animals, has an incidence of about 6%, in contrast to approximately 13% in American women (Abdelmegeed and Mohammed 2018; Gray et al. 2020; Dewi and Cline 2021). Furthermore, the lifespan of a canine is 10–13 years, shorter compared to humans, and half the duration of NHPs with a lifespan of 25–40 years. The shorter lifespan of canines compared to humans may allow for the earlier manifestation of epigenetic changes induced by exposure to bisphenol A, radiation, and workplace hazards, all of which can contribute to tumorigenesis. This combination of a shorter lifespan, similar tumor onset, and high CMT incidence not only provides a valuable model for discovering drug targets but also facilitates follow-up research on new cancer therapies (Owada et al. 2024).

CMTs exhibit molecular and pathological characteristics that align with human breast cancer subtypes (Canadas et al. 2019). In canines, hormone receptor expression is also common, especially in benign and early-stage malignant tumors, suggesting a similar hormone-dependent tumorigenesis (Klopfleisch et al. 2010). However, CMTs tend to lose hormone receptor expression as malignancy progresses, resembling aggressive human TNBC. HER2 + breast cancer, which responds to targeted therapies like trastuzumab, has been identified in both species, though HER2 overexpression in CMTs appears less frequent and less well-defined than in humans (Kwon et al. 2023). TNBC, known for its aggressive nature and lack of targeted therapies, has a strong counterpart in canines, where many malignant tumors exhibit low ER, PR, and HER2 expression, mirroring the poor prognosis of human TNBC (Namagerdi et al. 2020). A previous study suggested a molecular classification of CMT based on four hormone receptors and Ki-67 to define different subtypes, including luminal A, luminal B, HER2-positive, and TNBC in CMT (Varallo et al. 2019). The use of these markers demonstrated their ability to accurately define phenotypes of CMT for prognosis prediction. Similar to humans, the majority of CMT cases were classified as luminal A and luminal B (38.2% and 37.3%), indicating a favorable prognosis, followed by TNBC (15.4%) and HER2-positive (9.1%) cases, representing more aggressive features (Zappulli et al. 2015; Varallo et al. 2019; Gray et al. 2020).

There are many advantages to using companion animals to investigate cancer epigenomics; however, compared to the traditionally used experimental animals like mice in cancer research, there has been limited investigation. Companion animals are useful models for comparative medicine, bridging murine studies to human drug development.

## 3. Epigenetics in cancer therapy

Many Food and Drug Administration (FDA)-approved epigenetic drugs are primarily utilized for the treatment of leukemia and lymphoma (Nepali and Liou 2021). While DNA methylase inhibitors (DNMTi) and histone deacetylation inhibitors (HDACi) as standalone treatments have shown promise in pre-clinical studies in solid tumors, their clinical application and impact remain relatively limited. However, combining them with other cancer drugs has produced synergistic effects, enhancing treatment efficacy, reducing toxicity, and mitigating tumor resistance (Lu et al. 2020; Xu et al. 2022).

In this review, our focus will be on clinical and pre-clinical studies involving DNMTi and HDACi, which are among the most extensively utilized epigenetic modifiers in drug development. We will particularly emphasize their use in HBC, examining both monotherapy and combination chemotherapy with DNMTi and HDACi.

### 1) DNMT inhibitors

Three canonical isoforms have been identified in humans, namely DNMT1, DNMT3A, and DNMT3B, along with two non-canonical members, DNMT2 and DNMT3L. DNMT1 plays a critical role in maintenance methylation, while DNMT3A and DNMT3B are involved in de novo methylation, transferring a methyl group from S-adenosyl methionine (SAM) to the C-5 position of a cytosine residue (Vasanthakumar et al. 2013; Flesner et al. 2014; Yang et al. 2015). DNA methylation can be reversed or modified by enzymatic processes, specifically by dioxygenases like ten-eleven translocation (TET) proteins, which promote the oxidation of methyl-cytosine to hydroxy-methyl-cytosine during mammalian development (Taby and Issa 2010).

DNMT1/3A/B overexpression was observed in previous studies, indicating the clinical significance of these genes (Figure 2). DNMT3B overexpression is associated with reduced survival, lymph node metastasis, and worse prognosis in HBC patients (Butcher and Rodenhiser 2007). WIF1, as one of the tumor suppressor genes (TSGs), acts as a WNT pathway inhibitor, resulting in G1 arrest (Ai et al. 2006; Butcher and Rodenhiser 2007). The hypermethylation of WIF1 is carried out cooperatively by DNMT3B and DNMT1 (Ai et al. 2006). In the case of DNMT3A and DNMT1 overexpression led to hypermethylation of HOXA5, RARβ2, and RASSF1A (Bagadi et al. 2008). In ER- cell lines, the increased DNMT3A/B levels correlated with the hypermethylation of CXCL12, a chemoattractant for lymphocytes, megakaryocytes, endothelial cells, and stem cells. This hypermethylation potentially leads to interactions between HBC and other organs that express CXCL12, thereby contributing to tumor metastasis (Zhou et al. 2009). DNMT3A correlated with shorter OS, disease-free survival (DFS), brain metastasis, and hypermethylation of ER α and BRCA1 (Chen and Chan 2014; Yang et al. 2015). DNMT1 has been reported to affect the EMT program by up-regulating E-cadherin while down-regulating N-cadherin and vimentin, cooperatively with HDAC1/2 (Das 2023). In the case of CMT, the evaluation of aberrant DNMT expression is still scarcely known. To determine whether aberrant DNMT expression is associated with clinical features in CMT, similar to HBC, further studies are needed in a canine cohort.

DNMT inhibitors can be classified into two main categories: nucleoside analogs and non-nucleoside analogs (Table 2). Nucleoside analogs include compounds such as 5-azacytidine, 5-azadeoxycytidine, decitabine, guadecitabine, and zebularine. Non-nucleoside analogs are composed of small molecule agents that directly target the catalytic sites without integrating into DNA.

**Table 2.** Classification of DNA methyltransferases.

Epigenetic durg	category	Compound	Condition	Mechanism of action
DNMT inhibitor	nucleoside analog	5-azacytidine	MDS	Get incorporated in RNA and DNA
		5-aza deoxycytidine	MDS	
		Decitabine	MDS	
		Guadecitabin	MDS	Deoxyguanosine linked 5-aza-CdR
		Zubularine	MDA, AML	forms a covalent complex with DNMT
	non-nucleoside analog	Psammaplin	MDS	Hairpin loops and specific antisense oligonucleotides
		EGCG	MDS	Reduces DNMT expression
		Curcumin	AML	Polyphenolic compounds, induce global hypomethylation
		Hydralazine	MDS	DNMT1, DNMT3A inhibition
		Procainamide		DNMT1 inhibition
		MG98		Inhibits DNMT1 translation
		RG-108	Solid tumor	Blocks the DNMT1 active site
		SGI-1027		Induces DNMT1 degradation

DNMT: DNA methyltransferase; MDS: myelodysplastic syndrome; AML: Acute myeloid leukemia; EGCG: Epigallro Catechine Gallate.

5-Azacytidine (ZCyd) and 5-Azadeoxycytidine (ZdCyd) are involved in nucleoside analog DNMT inhibitors. 5-Azacytidine is incorporated into DNA and RNA during replication. Once integrated into the RNA or DNA strand via the ribonucleotide reductase pathway, it forms a covalent bond with DNMT enzymes, inhibiting their activity and preventing methylation of RNA or DNA (Tyagi et al. 2015). Both ZCyd and ZdCyd have received approval for the treatment of myelodysplastic syndrome (MDS) and acute myeloid leukemia (AML) (Schwartsmann et al. 1997; Momparler et al. 2014). While clinical studies on ZCyd have primarily focused on patients with MDS and AML, research is also ongoing on various other malignancies (Aparicio and Weber 2002; Linnekamp et al. 2017).

Azanucleoside-based therapies are currently undergoing phase I/II clinical trials in various cancer types, including solid tumors. DNMT inhibitors also hold strong potential for HBC therapy (Ren et al. 2018). In vivo studies, treatment with ZdCyd alters global DNA methylation, cell cycle and cisplatin sensitivity (Chekhun et al. 2016; Khan et al. 2017). A previous study explored the potential of DNMTi in targeting epithelial–mesenchymal transition (EMT) for the treatment of TNBC (Canadas et al. 2019). ZCyd treatment inhibits HBC brain metastasis by suppressing Wnt signaling, cell migration, invasion, and tumorigenesis in brain-colonizing cells (Klopfleisch et al. 2010).

In both humans and canines, ZCyd decreased in vitro growth, invasion, tumorigenicity, and mitochondrial activity while enhancing their susceptibility to apoptosis (Varallo et al. 2019; Namagerdi et al. 2020; Kwon et al. 2023).

Zebularine, a second-generation DNMTi, is an oral DNA-demethylating drug that has demonstrated stability in acidic environments as well as aqueous solutions it can also be incorporated into RNA or DNA (Barchi et al. 1995). Zebularine can induce the degradation of DNMT1, leading to further DNA demethylation. It induces apoptosis via the caspase 3/9 pathway and inhibits cell growth in SKBR3 cell lines (Eroglu and Celen 2019). Zebularine also effectively inhibited growth and induced dose-dependent apoptosis in lymphoid cells of canines (Flesner et al. 2014). Plasma pharmacokinetics and toxicity of zebularine in canines were estimated in 3 normal laboratory canines and 3 tumor-bearing canines (one invasive transitional cell carcinoma and two gastrointestinal stromal tumor canines) (Fulkerson et al. 2017). There were no zebularine-induced side effects such as hematologic abnormalities and dermatologic changes (Fulkerson et al. 2017). For optimal dosing interval, further investigation of aldehyde oxidase activity and half-life of zebularine incorporated into DNA would be required.

Decitabine, like 5-Azacytidine, is also approved by the FDA for the treatment of acute myeloid leukemia (AML) and myelodysplastic syndrome (MDS) (Schwartsmann et al. 1997). Decitabine inhibits DNA methylation in a dose-dependent manner. At a low dose, it can reactivate hypermethylated genes but reveals cytotoxicity in high doses (Momparler et al. 2014). Decitabine has demonstrated potential in reactivating hypermethylated genes and enhancing the doxorubicin sensitivity of HER2-positive HBC cells (Buocikova et al. 2022a). After exposure to DAC, the genes TET1 and TET2, which are involved in the oxidation of 5-methyl-cytosine, were found to be reduced, and several TSGs, including CDO1, CXCL12, ACHE, ITGA7 were up-regulated in JIMT-1 cell lines (Buocikova et al. 2022b). The combination therapy involving SAM and decitabine demonstrates enhanced anti-cancer efficacy in suppressing HBC growth and metastasis (Mahmood et al. 2020). The combination therapy of SAM and decitabine reduces mammary tumor volume and lung metastasis in a xenograft model (Mahmood et al. 2020).

Guadecitabine, also known as SGI-110, is a next-generation hypomethylating agent with potential applications in HBC treatment. Its unique composition as a dinucleotide of decitabine and deoxyguanosine linked by a phosphodiester bond offers advantages in terms of stability, pharmacokinetics, and administration route, which could make it a more convenient option for HBC patients. In the 4T1 and C57BL/6 J E0771 murine model, guadecitabine specifically targets excessive myelopoiesis within the bone marrow and significantly reduces MDSCs in the spleen, tumor, and blood. It can reduce systemic T-cell suppression, enhancing the host’s antitumor CTL response (Luker et al. 2020). Research on the responsiveness of guadecitabine in both human and canine cancers is still limited. Further studies are needed in the future.

Based on these pre-clinical data, to elucidate the therapeutic potential of DNMTi in HBC, clinical trials are currently underway, involving various combinatory therapies (Table 3).

**Table 3.** Ongoing clinical trials of Epi-drug.

Epi-drug	Drug combination	Status	Reference
Azacytidine	Monotherapy	II	NCT04891068
Azacytidine	Docetaxel/Paclitaxel/Irinotecan	I/II	NCT05381038
Decitabine	Doxorubicin/Carboplatin/Paclitaxel Pembrolizumab/Cyclophosphamide	I	NCT02957968
SAHA	Cedazuridine	I	NCT05673200
	Talazoparib/ASTX727	I	NCT04134884
	Olaparib	I	NCT03742245
	Carboplatin/Paclitaxel	II	NCT00616967
Abexinostat	Fulvestrant/Palbociclib	I	NCT04498520
Mocetinostat	Docetaxel	I	NCT00511576
Valproic acid	Monotherapy	II	NCT00395655
	Monotherapy	I	NCT01007695
	Monotherapy	I	NCT01808040
	Monotherapy	I	NCT01808040
	Temsirolimus	I	NCT01552434
	Monotherapy	I	NCT00020579
	Monotherapy	I	NCT02897778
Entinostat	Monotherapy	II	NCT03291886
	Anastrozole	II	NCT01234532
	Azacitidine	II	NCT01349959
	Exemestane/Goserelin/Goserelin Acetate	III	NCT02115282

Epi-drug: Epigenetic drug.

### 2) HDAC inhibitors (HDACi)

Aberrant expression of HDAC has been investigated in various cancers, including HBC. HDACi are primarily employed to target epigenetic modifications associated with cancer.

HDACi can be classified into four categories according to their chemical structures; short chain fatty acids, hydroxamates, cyclic peptides and benzamides (Bondarev et al. 2021). HDACi classification and their target are listed in Table 4. While HDAC inhibitors have demonstrated efficacy in treating MDS and AML patients, research in the context of solid tumors remains limited (San José-Enériz et al. 2019). In this review, we concentrate on in vitro and in vivo studies of HDAC inhibitors in HBC and their relevance to CMT.

**Table 4.** HDACi classification and their targets.

Epigenetic drug	category	Compound	Condition	Mechanism of action
HDACi	Short chain fatty acids	Valproic acid	TCL, Myeloma	HDAC I& II
	Hydroxamates	SAHA	Myeloma, Glioblastoma	Pan HDAC
		Belinostat		Pan HDAC
		ITF237		HDAC I& III
		4SC-201		Pan HDAC
		Panobinostat	Melanoma	Pan HDAC
		TSA	HBC, Leukemia	HDAC I& II
		Abexinostat		Pan HDAC
		Givinostat		Pan HDAC
		Tubacin		Pan HDAC
	Benzamides	Domatinostat		HDAC I
		Mocetinostat		HDAC I
		Entinostat	HBC, Lymphoma	HDAC I & III
	Cyclic peptides	Romidepsin		HDAC I

HDACi: Histone Deacetylase (HDAC) Inhibitor; TSA: Trichostatin A.

Suberoylanilide hydroxamic acid (SAHA, vorinostat) was classified as hydroxamate and pan HDACi. It was the first approved HDAC inhibitor for clinical use in T-cell lymphoma and multiple myeloma (Suzuki et al. 2022). Additionally, other clinical trials revealed SAHA’s therapeutic potential in solid tumors, including HBC (Ding et al. 2015). SAHA treatment resulted in a dose-dependent increase in apoptotic cells across all cell lines, with the most significant effect observed in T47D ER + cells, inducing cell cycle arrest in the G1 phase, especially in T47D (Zhao et al. 2014). However, A phase II trial to determine the response rate of SAHA mono treated in patients with metastatic HBC, identified that vorinostat alone did not meet the RECIST response criteria for sufficient single-agent efficacy (Vansteenkiste et al. 2008). In a phase II study, virinostat combined with tamoxifen treatment in hormone receptor-positive HBC patients identified that combined treatment leads to tumor regression or extended disease stabilization in 40% of individuals who had previously experienced progression despite prior hormonal therapy and chemotherapy (Munster et al. 2011). In canine solid tumors, SAHA revealed antitumor effects in osteosarcoma (OS) cell lines, inducing apoptosis by dose-dependent manner and urothelial carcinoma cells, inducing growth inhibition and cell cycle arrest (Munster et al. 2011).

Panobinostat, classified hydroxamate and pan HDACi developed by Novartis, is used for the treatment of multiple melanomas (Garnock-Jones 2015). Pre-clinical studies on HBC revealed that Panobinostat stimulated exosome release via Vps34/Rab5C pathway, inducing autophagy in HBC and significantly reducing the expression of EMT-associated genes, including p-MEK, p-ERK, Snail and Vimentin (Wang and Yin 2023). In TNBC, Panobinostat treatment induced ROS-dependent ER stress, enhancing the apoptotic population compared to the control. Furthermore, Panobinostat reduces deacetylation and hyperacetylation in H3 (Lys9) and H4 decreasing proliferation, survival and G2/M cell cycle arrest in TNBC cell lines. In canine hematological tumors, Panobinostat also inhibits tumor growth and induces apoptosis by histone H3 acetylation (Tate et al. 2012).

Trichostatin (TSA) is classified as hydroxamate, which specific inhibitor of mammalian HDAC I and II, inducing cancer cell apoptosis. In ER-positive HBC, TSA treatment induces reverse EMT via EMT marker, SLUG suppression (Wang et al. 2020b). After treatment of TSA, decreased expression of cell cycle regulators such as cyclin D1, CDK4, and CDK6 and increased pro-apoptotic molecule, BCL-XL (Wang et al. 2020b). Similarly, TSA treatment induces sub G1 arrest and apoptosis in grade 3 canine mast cell tumors and cell cycle inhibition and anti-proliferation in canine mammary tumors (Bondarev et al. 2021).

Valproic acid, involved in short-chain fatty acids also FDA approved HDAC 1 and II inhibitor. Valproic acid is clinically used in epilepsy, bipolar disorder and migraine patients and extensive clinical trials have been conducted in cervical cancer, ovarian cancer, HBC, AML, and MDS (Tandon et al. 2016; Li et al. 2021). In virto studies identified that Valproic acid decreased the Warburg effect by down regulation of pyruvate kinase M2 isoform (PKM2) in both MCF7 and MDA-MB-231 (Li et al. 2021). In another study, valproic acid reduced proliferation in MCF7, SKBR3, BT-474 and MDA-MB-231. In HER2+ HBC valproic acid also anti-cancer effect by inducing cell cycle arrest by increasing p21 WAF1 and apoptosis through acetylation of HSP70 (Mawatari et al. 2015). The pharmacodynamic effects are already conducted in 21 canine spontaneous OS. All doses used in the dose escalation trail were tolerated and serum valproic acid level increased linearly. There was hyperacetylation in OS tissue peripheral blood mononuclear cells without myelosuppression (Wittenburg et al. 2010).

Entinostat is an oral bioavailable drug classified as benzamide, inhibiting HDAC I and III. It works with multiple myeloid malignancies (Tandon et al. 2016). Clinical trials of the use of entinostat have been conducted in Hormone Receptor (HR) positive advanced HBC, lymphoma, non-small cell lung cancer, and colorectal cancer (Karasic et al. 2022; Gentzler et al. 2023; Xu et al. 2023). Phase I trial of entinostat in HR + metastatic HBC was conducted to evaluate the efficacy, pharmacokinetics and safety of entinostat. Entinostat is well tolerated at all doses serum levels increased linearly (Wang et al. 2021). In phase III clinical study conducted in HR + patients with recurrence after endocrine therapy, identified combination therapy with entinostat and exemestane showed an improved progression-free survival (PFS) compared to exemestane monotherapy (Xu et al. 2023).

The anti-cancer effect of HDAC inhibitors was also identified in canine urothelial carcinoma cells (cUC), OS, prostate cancer (PCa) and hemangiosarcoma (HSA) (Elshafae et al. 2017; Murahari et al. 2017; Eto et al. 2019; Suzuki et al. 2022). SAHA induces histone acetylation of cell cycle-related genes, consequently affecting cUC cell growth inhibition and G0/G1 cell cycle arrest (Eto et al. 2019). SAHA also activates inflammatory cytokines and induces apoptosis, while it does not affect tumor growth inhibition (Suzuki et al. 2022). AR-42, another histone deacetylase inhibitor, has been reported to decrease proliferation, invasion, migration, and expression of telomerase, and induce apoptosis in canine PCa (Elshafae et al. 2017). Hence, AR-42 reduces OS cell viability by attenuating AKT activation and reveals a synergistic effect in combination therapy with doxorubicin, reducing their combination index (CI) value (2.6–4.57 fold according to the cell lines) (Murahari et al. 2017). Diverse HDACi clinical trials are ongoing; the details are listed in Table 3.

## 4. Conclusion

This review highlights the advantages of using companion animals, particularly canines, to better understand epigenetic modification in HBC. Approximately half of the female canines spontaneously develop mammary tumors, which closely resemble HBC in terms of histopathology, clinical manifestation, metastasis, recurrence, genetic predisposition, and treatment response or resistance patterns. Notably, the spontaneous development of mammary tumors in canines offers a natural disease model that avoids the ethical concerns associated with induced cancer models. This natural occurrence also facilitates the study of environmental influences and epigenetic alterations, which are often difficult to replicate in other animal models. Furthermore, canine models provide significant advantages for testing the efficacy of new-generation epigenetic drugs in humans, monitoring responses to antitumor therapy, and assessing the impact of drug delivery systems under conditions reflective of human biology.

In conclusion, this review underscores the growing importance of epigenetic therapy and comparative medicine as a valuable approach to cancer treatment and prevention while also acknowledging the need for further research and development to address existing limitations and challenges in this field.
